# Spontaneous rupture of the right gastroepiploic artery: unusual cause of acute abdomen and shock

**DOI:** 10.1186/1749-7922-4-24

**Published:** 2009-06-17

**Authors:** Karim Ibn Majdoub Hassani, Ali Bounekar, Jean-Manuel Gruss

**Affiliations:** 1 Department of General Surgery. Universitet Hospital Hassan II. Fes. Morocco; 2 Department of General Surgery. Inter Communal Hospital Villeneuve Saint George. France

## Abstract

**Introduction:**

Spontaneous rupture of the right gastroepiploic artery is an extremely rare case which can be a cause of abdominal apoplexy.

**Case report:**

We present a case of a 64-year old woman with a Spontaneous rupture of the right gastroepiploic artery with hemorrhagic shock that was successfully treated by emergency surgery.

**Conclusion:**

Simultaneous restoration of circulating volume and rapid diagnosis are keys in determining the patient outcome in this situation. Though the mortality is high if untreated, the operation is relatively simple and carries a low risk.

## Introduction

Spontaneous rupture of the right gastroepiploic artery is an extremely rare case which can be a cause of abdominal apoplexy, and which should be considered in the differential diagnosis of unexplained hemorrhagic shock and if hemoperitoneum is encountered while performing a laparotomy. Simultaneous restoration of circulating volume and rapid diagnosis are keys in determining the patient outcome. Though the mortality is high if untreated, the operation is relatively simple and carries a low risk.

## Case report

A 64-year old woman was presented to the emergency department with acute abdominal pain and breathlessness of which she was suffering few hours before her presentation to the emergency room. Her medical history revealed recurrent upper abdominal discomfort over the last 4 months, and did not suggest any major disease except hypertension, that she has been treating since seven years. Besides, she had no prior history of abdominal surgery or trauma.

The physical examination revealed a conscious woman with discolored conjunctives and severe cutaneous paleness, shortness of breath, tachycardia with a weak and rapid pulse rate of 126 beat per minute, and hypotension with a systolic blood pressure of 80 mmHg. At the abdominal examination, there was a general abdominal tenderness.

Laboratory data showed a white blood cell count of 7 900/mm3, a hematocrit of 18%, and serum hemoglobin concentration of 6,1 g/dl, with a normal blood platelet level (390 000/mm3), a blood urea of 0,25 g/L, and a creatinine level of 10 mg/L. Hemostasis laboratory data, chemistry and Serum lipase were within normal limits.

**Figure 1:**
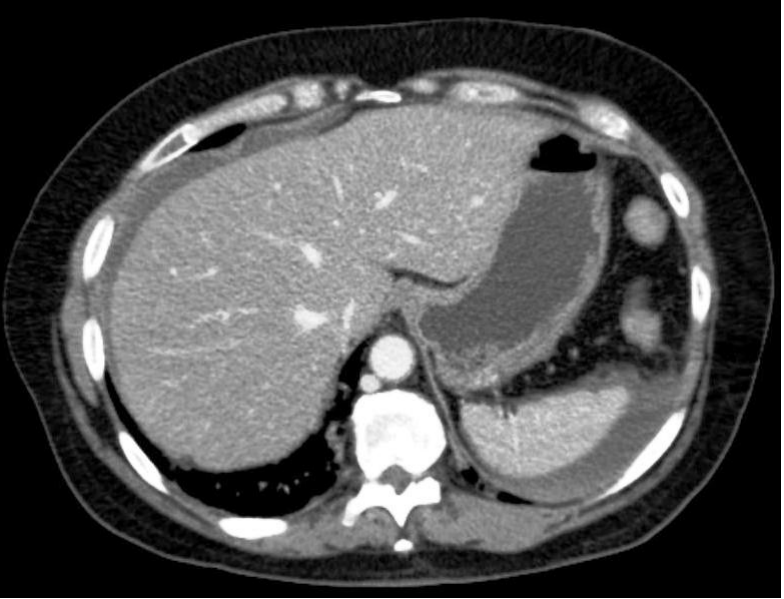
**Abdominal computed tomography (CT) scan (axial) with intravenous contrast demonstrating an important hemoperitoneum with densities around the spleen and the right lobe of the liver**.

**Figure 2:**
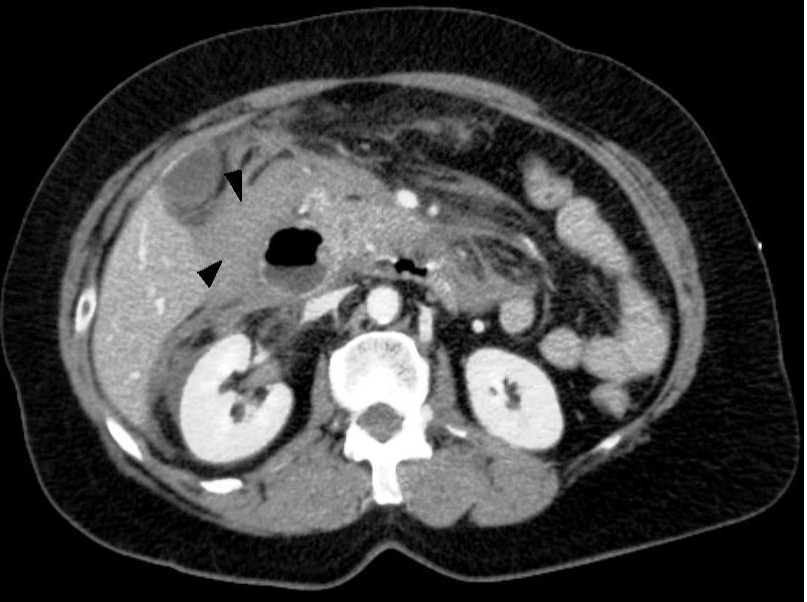
**Abdominal CT (axial) with contrast demonstrated a large density around the duodenum, the fluid densities were felt to represent a hematoma**. (Black arrowhead).

**Figure 3:**
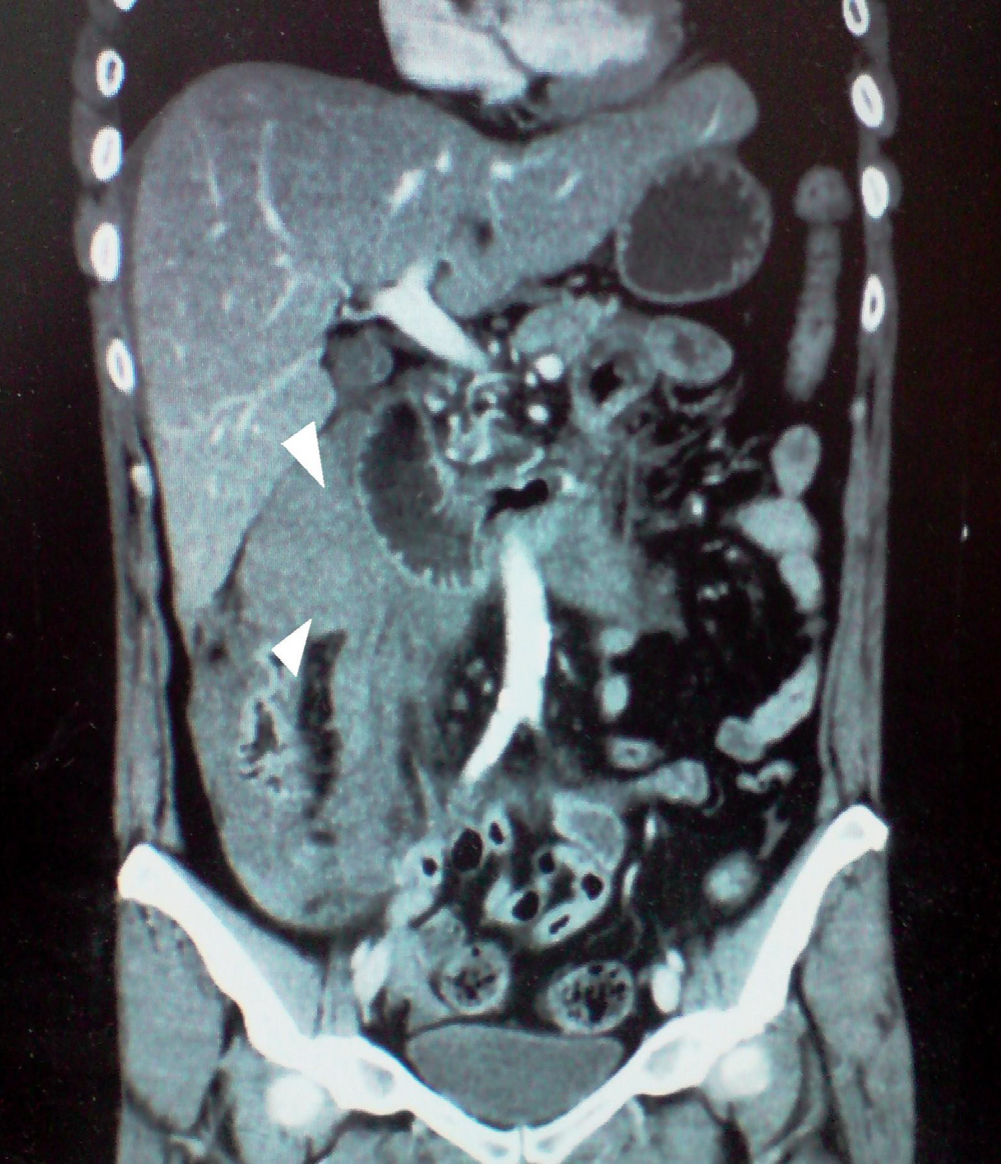
**Paraduodenal hematoma shown in the coronal Abdominal CT with contrast**. (White arrowhead).

The patient was shift to the intensive care unit (ICU) with a swift assessment of her airway, breathing and circulation. The initial resuscitation was begun by physiological serum and conventional crystalloid solutions; then she was transfused by 8 units of red blood cells. After hemodynamic stability, an abdominal computerized tomography (CT) was performed and revealed the presence of an important hemoperituneum with two fluid densities around the spleen and the liver [Figure 1], it also revealed a large density around the duodenum which represented a hematoma [Figure 2, 3]. There was no free air and all solid organs had a normal appearance.

It was impossible to obtain the opinion of either a vascular surgeon or an interventional radiologist for this acute intraabdominal hemorrhage, and it was indispensible to shift the patient to the operating room for an emergency surgery to control the source of bleeding. An emergency exploratory laparotomy was performed under general anesthesia. This Surgical exploration showed an important hemoperituneum and a large periduodenal hematoma which was extending into the retroperitoneal space. Two liters of blood were evacuated from the free peritoneal cavity. Besides, we noted a significant bleeding from the right gastroepiploic artery, with no obvious aneurysm, that was successfully ligated. Further exploration identified no additional bleeding, and the retroperitoneal hematoma was respected. The patient recovered well without postoperative complications and she was discharged 5 days after the surgery.

## Discussion

Idiopathic spontaneous intraperioneal hemorrhage (ISIH) was first reported by Barber in 1909 and was later termed "abdominal apoplexy" by Green and Powers in 1931. Its true incidence is unknown [1]. Intra-abdominal hemorrhage may be secondary to blunt trauma, aneurismal rupture (central or visceral), solid organ malignancy (hepatic or renal), or inflammatory erosive processes (pancreatitis or pseudo cyst). It may be idiopathic, as well [2]. Bleeding may be intraperitoneal or retroperitoneal, and is frequently found in conjunction with hypertension (33–50%) and atherosclerosis (80–87%) [1-5]. Rupture with subsequent hemorrhage in the absence of abdominal trauma is exceedingly rare, even if 30% of cases historically have no identifiable source [3]. Aneurysms of the visceral circulation are rare, accounting for 0.1–10.4% in autopsy statistics [4,5]. The splanchnic vessels most commonly involved are the splenic (56%), hepatic (19%), superior mesenteric (8%) and gastric (5%) [1]. The incidence of a gastroepiploic artery rupture is rare, account for 4.5% of the overall splanchnic origins of idiopathic spontaneous intraperioneal bleeding [6,7].

Spontaneous nonaneurysmal right gastroepiploic artery rupture (RGEA) is among the rarest [1]. None of the reviewed reports have dealt with, specifically, right gastroepiploic artery rupture without aneurismal changes [1]. The previous enigmatic 20–30% of apoplexy with no identifiable source is now thought to be related to common vascular disease with arteriosclerosis and hypertension felt to represent risk factors [8]. The exact mechanism is unknown, but likely represents weakness of the tunica media, predisposing rupture in the face of abrupt increases in pressure. Pathology specimens regularly exhibit disruption of elastic lamellae [9,10]. Unfortunately, we didn't have any histopathology of the vessel wall to know the exact etiology of our patient's disease; however we think that the data above is the main cause of her RGEA rupture especially that she has been treating hypertension for seven years and also because the surgical exploration didn't reveal any evident aneurysm of the RGEA. Spontaneous hemorrhage can be seen with inflammatory erosive processes which explain the association with necrotizing arteritis in polyarteritis nodosa and rheumatoid arthritis [8,9]. This may explain that an aneurysmic stage does not necessarily precede the spontaneous rupture of a visceral artery [1].

The presentation and clinical progression of abdominal apoplexy frequently follows a rather predictable course. Before rupture, there may be a history of vague abdominal pain which is the case of our patient. The symptoms are usually non specific. Physical examination before or soon after rupture is likely to be relatively normal although no one finding is pathognomonic. Hypotension may be present depending on whether the hemorrhage is contained or free intra-abdominal rupture exists. The presentation of acute hemoperitoneum is divided into three main phases: an early phase of mild-to-severe abdominal pain, a latent phase lacking any symptomatology, lasting from hours to days and a final phase of acute hemoperitoneum in which the patient experiences a rapid increase in the severity of the symptoms, especially the abdominal pain [1]. The diagnosis is generally made on laparotomy for haemodynamic instability which is the case of our patient. In less urgent cases, ultrasonography or CT scan with intra venous contrast can be used. In the hemodynamically unstable patient, FAST (focused assessment by sonography in trauma) examination may be useful to detect intra-abdominal hemorrhage. However, CT scan represents the most important imaging technic. The use of intra venous contrast is currently recommended if the patient is stable enough for the delay associated with administering oral contrast. CT angiography of vessels has proven useful as a screening tool using small amounts of contrast to elucidate sites of active bleeding [11,12]. Treatment of spontaneous intraperitoneal bleeding, as with other bleeding phenomena, revolves around resuscitation and restoration of circulating volume. This has traditionally been followed by surgical correction. The surgical management consists of resection of the aneurysm, ligation of the feeding vessels or some forms of arterial reconstruction [5,13]. Radiological intervention with embolisation of the feeding vessel is an option in splanchnic aneurysms. A research of the literature revealed that ligation of vessels with or without resections is the preferred option, as this is relatively simple and carries a low risk [11]. Non-surgical mortality has historically approached 100%. Reported mortality with non-therapeutic exploratory laparotomy varies from 40% to 66%. Surgical ligation represents a well-studied definitive treatment, reducing mortality to 8.6%. After ligation there are no reported recurrences [9].

## Consent

Written informed consent was obtained for publication of this case report and accompanying images. A copy of the written consent is available for review by the Editor-in-Chief of this journal.

## Competing interests

The authors declare that they have no competing interests.

## Authors' contributions

KI is a surgeon who was drafting the manuscript and revising it critically for content and was involved in literature research. AB and JMG were surgeons treating of the patient and were involved in revising the draft critically for content. All authors read and approved the final manuscript
